# Speech-aid prosthesis in velopharyngeal incompetency patient with cleft palate: can speech aids be applicable for adult patient?

**DOI:** 10.1186/s40902-021-00315-5

**Published:** 2021-08-09

**Authors:** Dong-Cheol Kang, Jung-Ho Park, Hyun Seok, Jin-A Baek, Da-Wa Kim, Seung-O Ko

**Affiliations:** 1 Department of Oral and Maxillofacial Surgery, School of Dentistry, Jeonbuk National University Dental Hospital, 20, Geonji-ro, Deokjin-gu, Jeonju-si, Jeollabuk-do Republic of Korea; 2 Speech Language Clinic, Department of Oral and Maxillofacial surgery, School of Dentistry, Jeon Buk National University, Jeon Ju, Republic of Korea

**Keywords:** Cleft palate, Speech, Adult, Pharyngeal

## Abstract

**Background:**

Velopharyngeal incompetence (VPI) therapy for cleft palate (speech therapy alone, speech therapy using speech aids, or combined therapy such as speech therapy using a pharyngeal flap), is more effective in younger patients than in adult patients. Speech therapy is known as very difficult for patients who still have VPI as an adult. Because of the possibility of subsequent speech disorders, the timing of surgery for cleft palate is accelerating. Herein, we present a case of an adult with articulation disorder due to VPI who was treated by speech therapy and a speech-aid prosthesis.

**Case presentation:**

A woman who underwent cleft palate surgery at 8 years of age still had difficulty with articulation due to VPI as a 24-year-old adult because of a lack of continuous speech therapy. We decided to use a speech-aid application using palatal lift, and a reduction program was conducted four times, along with simultaneous speech therapy, over a period of 1 year and 7 months. During the therapy period, she was able to speak normally within a relatively short period of time, and after implementation of the reduction program, the therapy was completed by completely removing the device. Long-term observations have shown normal speech function without recurrence, even after the device was removed.

**Conclusion:**

As seen in this case, speech therapy using speech aids can show a good result for adult patients with cleft palate who missed the usual timing for the treatment of articulation disorders, depending on the situation. Therefore, it is hereby reported as a therapy option worthy of consideration.

## Introduction

Submucous cleft palate (SMCP), a type of cleft palate, is a congenital disease caused by abnormal development of the soft palate muscle tissue. Such pathological conditions of the cleft palate may cause functional problems in various muscles of the nasopharynx (including the tensor veli palatini, levator veli palatini, palatopharyngeus, palatoglossus, uvulae, salpingopharyngeus, and pharyngeal constrictor muscles), potentially resulting in velopharyngeal incompetence (VPI) and articulation disorder.

Articulation disorders are usually treated with clinical methods such as surgical therapy (pharyngoplasty), electrotherapy, and speech therapy. Speech aids can also be used and are often effective when used together with basic clinical methods. When speech aids are applied to children and adolescents with articulation disorder and history of cleft palate, the prognosis is good; however, the prognosis is uncertain when applied to adults.

Herein, we report a case of an adult with articulation disorder and a history of cleft palate who underwent treatment with both speech therapy and a speech-aid prosthesis. She completed treatment, with good outcomes at follow-up.

## Case presentation

A 24-year-old woman visited our Oral and Maxillofacial Surgery department due to discomfort in talking (e.g., mutacism). She wished to determine whether there was a problem with her oral structure and whether speech therapy was required. She had a history of SMCP. She underwent palatorrhaphy under general anesthesia at about 8 years of age, after having lost an opportunity to undergo surgery at 1 year of age. She received speech therapy for approximately 6 months postoperatively at the hospital of the surgery. However, due to personal problems, she was unable to undergo additional treatment since that time.

She completed speech evaluation tests before and after surgery at 8 years of age and before further treatment at the age of 24 years. An intraoral examination was conducted, and subjective evaluations included the Peabody Picture Vocabulary Test (PPVT) and VPI articulation screening test. Additionally, objective evaluations, such as the Nasalance test (using a Nasometer 6200 II), were used as speech evaluation tools. The classification devised by Shin et al. [1] was used for the nasality test (Table 1).

**Table 1:** Nasalance classification by Shin’s criteria [1]

20% below	Normal
30–35%	Mild nasality
35–45%	Moderate nasality
45–60%	High nasality
60% above	Severe nasality

### Preoperative evaluation

The results of the preoperative evaluation were as follows. On oral facial examination, a bifid uvula was observed, lip protrusion was incomplete, and the range of tongue movement was limited. When the tongue was stretched downward, it bent, creating a flection. There was a dint at the front part of the tongue. On the PPVT, the equivalent age was 7 years and 8 months; thus, her receptive language vocabulary was delayed by about 7 months compared to her chronological age of 8 years and 3 months. On the VPI articulation screening test, the intelligibility of perfect articulation was 59.1%. The main errors were due to sound distortions caused by hypernasality, nasal snorting, and nasal emission. Among all the patterns, a distortion pattern (velar consonant /k/, hard palatal affricate /c/, /c’/, /c^b^/, alveolar fricative /ʃ/) accounted for 75% of errors, and a substitution pattern (alveolar fricative /s/, /s’/ -> alveolar plosive /tb/, /t’/) accounted for 25%. On the Nasalance test, the velopharyngeal closure function was inadequate. Vowels /a/, /e/, /ja/ had moderate nasality; vowels /o/, /u/, /je/, /wi/, with no nasal passage had high nasality; and the vowel /i/ had severe nasality.

### Postoperative evaluation

The results of the postoperative evaluation were as follows. On oral facial examination, the postoperative state of the bifid uvula was observed. The movement range was limited in the case of tongue elevation. When the tongue was stretched downward, the tongue was observed as stressed and bent. On the VPI articulation screening test, the intelligibility of perfect articulation was 68.2%. The main errors were due to sound distortions caused by hypernasality, nasal snorting, and nasal emission. Among all patterns, a distortion pattern (palatal affricate /c/, /c’/, /c^b^/, alveolar fricative /ʃ/) accounted for 62.5% of errors, and a substitution pattern (alveolar fricative /s/, /s’/ -> alveolar plosive/tb /, /t’/) accounted for 37.5%. On the Nasalance test, the velopharyngeal closure function was inadequate. The nasality level had slightly decreased compared to the preoperative level. Vowels /a/, /e/, /je/, with no nasal passage had moderate nasality. Vowels /i/, /o/, /u/, /wi/ had high nasality.

### Comparison between before and after surgery

Comparing the patient’s preoperative and postoperative articulation accuracy, the accuracy was observed to decrease due to errors from sound distortions caused by hypernasality and severe nasal snorts (Table 2). Furthermore, based on the results of the Nasalance test, the velopharyngeal closure function was confirmed as inadequate, but nasality was slightly reduced after surgery (Tables 3, 4, and 5).

**Table 2:** Velopharyngeal incompetence articulation screening test results

	Pre-Op		Post-Op	
Perfect articulation rate	59.1%	(13/25)	68.2%	(15/25)
Error patterns	Distortion	75.0%	Distortion	62.5%
	Substitution	25.0%	Substitution	37.5%

*Op* operative

**Table 3:** Nasometer II test results for words

	a	i	E	O	u	ja	je	wi
Pre-Op	41.78	90.52	46.03	68.47	56.95	48.06	65.32	68.86
Post-Op	39.86	65.25	42.02	57.23	53.06	13.06	32.83	52.32
Before SAP	22	42	6	13	8	6	15	25

Units: %

Nasalance was measured three times: before surgery and after surgery at 8 years of age and at 24 years of age

*Op* operative, *SAP* speech-aid prosthesis

**Table 4:** Nasometer II test results for sentences

Reading passage	Pre-Op	Post-Op	Before SAP
Sea passage	56.26	69.65	31
Rabbit passage	32.21	47.17	35

Units: %

Nasalance was measured three times: before surgery and after surgery at 8 years of age, and at 24 years of age

*Op* operative, *SAP* speech-aid prosthesis

**Table 5:** Nasometer II classification results for words

	A	I	E	O	u	ja	je	wi
Nasalance	22	42	6	13	8	6	15	25
Classification	Mild	Moderate	No	No	No	No	No	Mild

Units: %

### Speech therapy and speech-aid prosthetic treatment

**Fig. 1:**
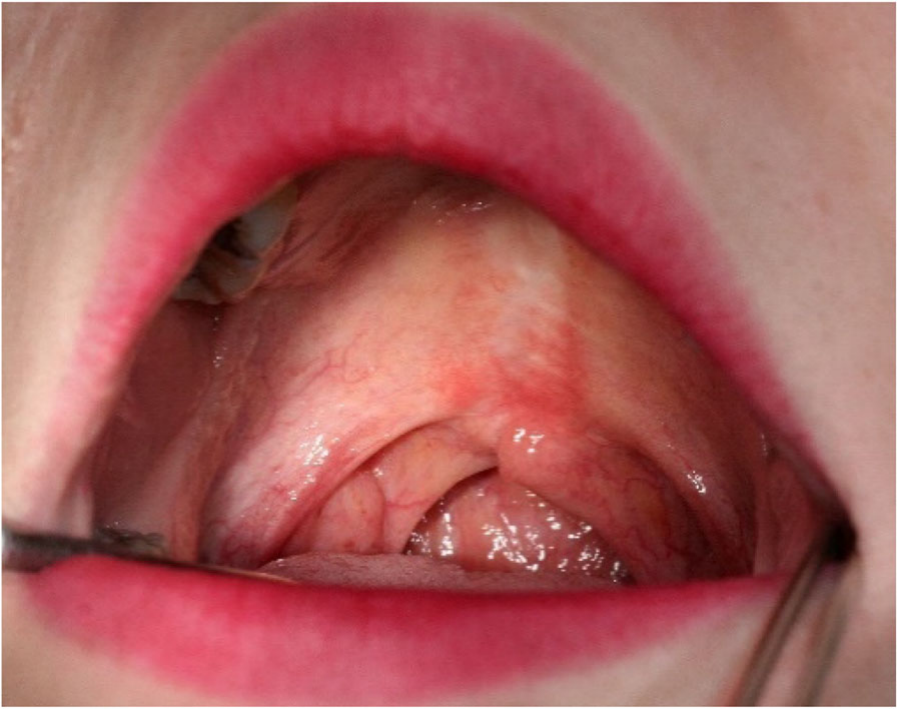
Intraoral view. A scar on the soft palate from the palatorrhaphy is observed

About 15 years after surgery, she underwent a speech evaluation at our hospital, and treatment with speech therapy and speech aids was started to improve her mispronunciation symptoms. At the first examination, a palatorrhaphy scar related to a history of cleft palate was observed in the oral cavity. In addition, there were no specific findings (Fig. 1). On the articulation examination, the consonant accuracy was 86.05%, and the main errors were a slight distortion in the alveolar (ㅅ, ㅆ) and palatal consonants (ㅅ, ㅆ). The Nasalance test showed moderate nasalance for vowel /i/, and mild nasalance for /a/ and /wi/ (Table 5).

**Fig. 2:**
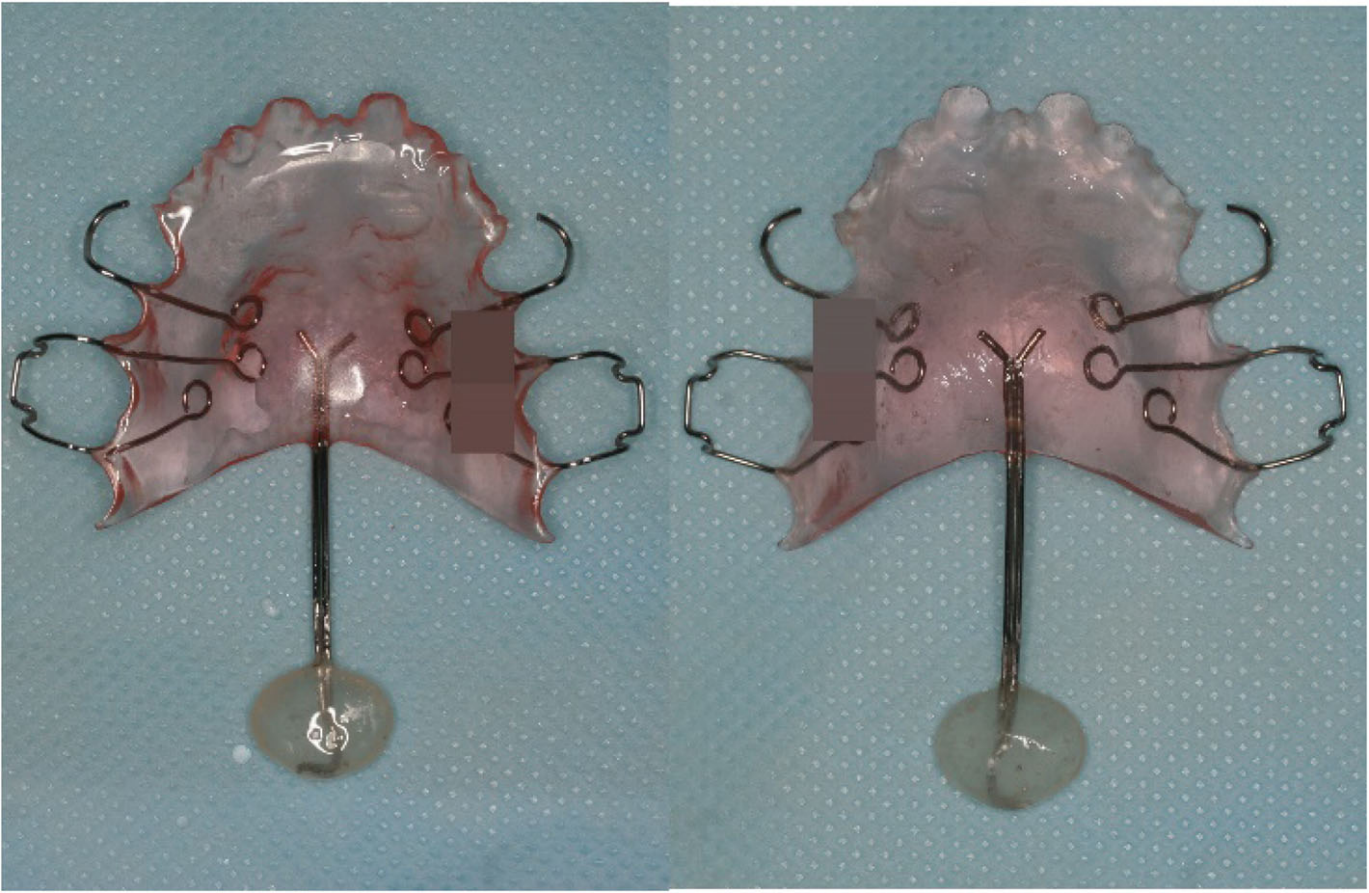
Palatal lift speech aids. Palatal lift was selected as a speech aid

**Fig. 3:**
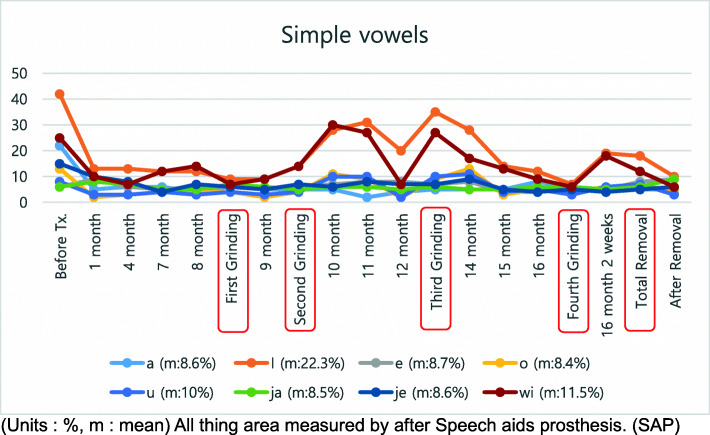
Nasalance for simple vowels. All object areas were measured after SAP therapy. m, mean; SAP, speech-aid prosthesis

**Fig. 4:**
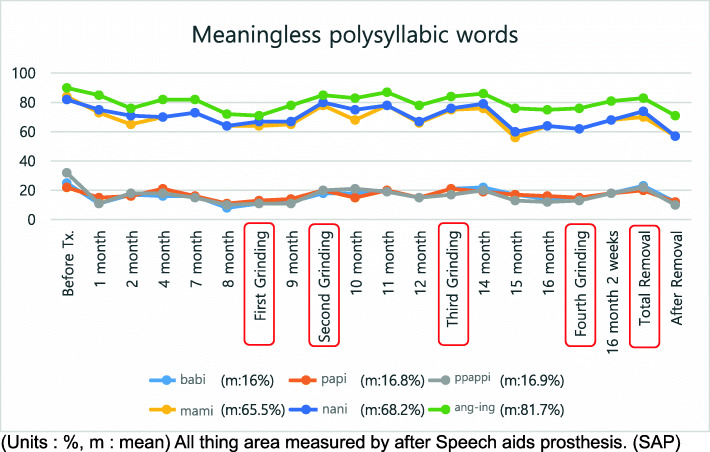
Nasalance for meaningless polysyllabic words. All object areas were measured after SAP therapy. m, mean; SAP, speech-aid prosthesis

**Fig. 5:**
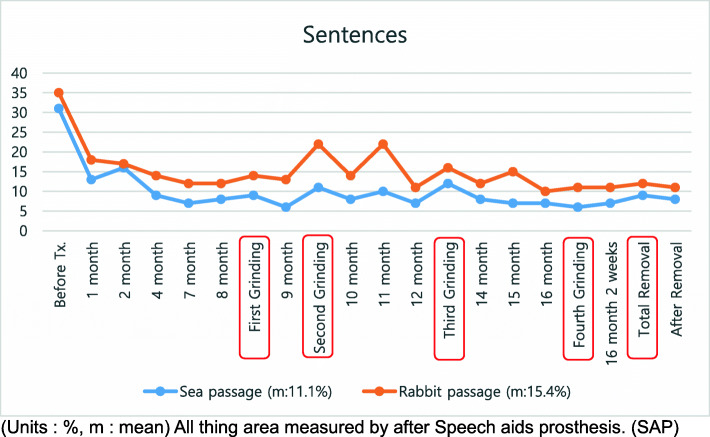
Nasalance for sentences. All object areas were measured after SAP therapy. m, mean; SAP, speech-aid prosthesis

A treatment plan was established by combining speech therapy with speech aids. For the speech-aid appliance, a palatal lift (Fig. 2) was manufactured. Speech therapy was administered once a week. Nine months after treatment, she showed stable nasality overall. Since no specific findings, such as hypernasal sounds were observed, the appliance was reduced. While observing the progress, this process was performed 4 times in total. The treatment took about 1 year and 6 months. During the treatment period, nasality evaluations and speech tests were performed continuously, along with appliance treatment (Figs. 3, 4, and 5). The speech samples used for the nasality evaluation were as follows: vowels: /a/, /i/, /e/, /o/, /u/, /ja/, /je/, /wi/ (Fig. 3); meaningless polysyllabic words: /babi/papi/, /ppappi/, /mami/, /nani/, /ang-ing/ (Fig. 4); and sentences: Sea passage (no nasal passage): “I’ll go to the beach on Monday afternoon to catch clams and shrimps, and come back early in the morning on Tuesday.”, and Rabbit passage (no nasal passage): “Let’s open the book together. It is the running story of a turtle and a rabbit. The rabbit shouted loudly to the turtle to have a race, and the turtle said yes” (Fig. 5).

## Discussion

The primary function of velopharyngeal action is to achieve normal pronunciation by promoting closure between the nasal cavity and the oral cavity. In this moment, the soft palate is located in the posterior lower part of the hard palate in a halting state, keeping the mouth and nasal cavity open. When nasal sounds are produced, airflow from the lungs and voice passes through this space, resulting in pronunciation. However, the mouth cavity and nasal cavity are completely closed because of the soft palate and the sphincter of the pharynx rear wall and side wall, when using other functions involving the oral cavity, such as making oral sounds, swallowing, blowing, and sucking. In the oral cavity, the middle 1/3 of the soft palate moves posteriorly upward, the pharynx rear wall moves forward, and the pharynx side wall moves inward to close the nasopharynx during such functions [2].

If there is a problem with the velopharyngeal action, the closing function of the velopharyngeal valve becomes incomplete, causing incomplete functions during actions such as vocalization and swallowing. Therefore, VPI, as velopharyngeal dysfunction, is a case in which the pharyngeal wall is not closed because the soft palate is short. One of the causes of VPI is a cleft palate. Patients with a cleft palate usually undergo surgical treatment (palatorrhaphy) at a young age [3]. However, even after surgery on the cleft palate, VPI may remain in 10–20% of cases. Characteristic speech disorders, such as resonance, voice, and articulation disorders, appear due to abnormal forms of the articulation organs, VPI, etc. [4].

Due to various speech disorders observed in patients with VPI, such patients show certain speech phenomena. First, voice energy is leaked into the nasal cavity, causing hypernasal sounds, and changes in speech habits are produced as compensatory actions. Vocalization, resonance, and breathing were all affected. Substitutions, distortions, and phenomena for aspirated sound, glottal stop, and pharyngeal fricative appear, which significantly reduces the patient’s speech intelligibility [5].

In patients with VPI, various treatment methods can be attempted, with 3 main types: (1) speech therapy, (2) surgery, and (3) speech-aid treatment. In patients with VPI, speech therapy is a basic treatment, and additional surgical methods and speech-aid treatment should be considered as supplementary means [6]. In the current case, the patient underwent palatorrhaphy at about 8 years of age due to severe nasal sounds and articulation disorder caused by an incomplete cleft palate. However, there was no subsequent continuous follow-up, and approximately 15 years passed without speech therapy.

Even after the patient became a 24-year-old adult, her pronunciation was still observed as difficult. After performing various examinations for articulation disorders, such as intraoral and extraoral tests and speech tests, VPI correction was thought to be the only remaining method. Thus, we decided to perform speech therapy and speech-aid treatment, without surgery. Furthermore, a previous study showed good results with a preservative method in the short-term, without surgery, when using speech therapy and pronunciation aid in patients with a history of SMCP and showing VPI [7].

There are two types of speech-aid appliances: palatal lifts and speech bulbs for the soft palate. Palatal lift of the soft palate can be used in patients who have normal palatal shape but suffer from VPI with paralysis or partial paralysis in the soft palate. The speech bulb is mainly used in patients with anatomically abnormal soft palates, such as a cleft palate or short soft palate [8]. It has been reported that the therapeutic effect of these speech aids (palatal lifts and speech bulbs) is lifting the soft palate, closing the palatopharyngeal gap, and facilitating palatopharyngeal activity and pharyngeal muscle contraction [9]. The advantages of speech aids applied to patients with VPI are as follows: they do not damage the nasopharynx area at all, they artificially improve VPI by using the patient’s own nasopharynx sphincter function, they can be reproduced, and it is possible to improve the overall VPI function by attaching the most suitable appliance to the nasopharynx during functioning (pronunciation).

Kipfmueller and Lang [10] reported on the effectiveness of speech aids for speech intelligibility in 40 patients and confirmed that the appliance improved articulation disorder in patients with VPI. In addition, Israel et al. [11] observed the effect of speech correction in patients with VPI using speech aids by applying the prosthesis to approximately 400 patients. After 3–5 years of treatment, 25–45% of patients showed normal speech function even after the speech-aid prosthesis was completely removed [11]. Wolfaardt et al. [12] performed treatment using a speech-aid appliance in 32 patients, and 21 (66%) patients showed improvement in their articulation disorder. Among these, 14 (67%) patients could pronounce normally even after complete removal of the speech-aid appliance [12]. Yoon et al. [13] reported that speech therapy performed in adult patients using palatal lift resulted in a significant decrease in nasality and an increase in speech intelligibility.

When patients who underwent surgery due to cleft palate continue to show articulation disorder caused by VPI, they can show normal speech function after a removal program without any equipment if they are treated with a speech-aid appliance at the age of 6–12 years, when the soft palate length and muscle movement are relatively adequately maintained [14]. After a certain period of time has passed after the application of a speech-aid appliance, normal conversation is possible in 30% of patients, even if the equipment is removed. Additionally, the younger the patient, the better the outcome [8].

However, Shin and Ko [8] evaluated the treatment effect of a speech-aid appliance in 7 patients (one youth and 6 adults) and reported that nasality was significantly decreased after appliance application relative to the before-application level [8]. In particular, 6 adult patients (over 20 years of age) with speech aids showed higher nasality in the sentence pattern of hypernasal sound than that for normal individuals before the appliance application. However, at 3 months after wearing the appliance, all levels decreased to within the normal range, with the exception of a slight hypernasal sound at the high vowel /i/. Therefore, it was concluded that the VPI function recovered to almost normal levels at 3 months after applying the speech-aid appliance [8].

In the current case, a patient who underwent surgery due to cleft palate was treated with a speech-aid prosthesis in adulthood, but not in childhood or adolescence. This patient underwent soft palatal lift and received speech therapy once a week. The patient started to activate the functional part after 1 week of adaptation after the application of the soft palate lift. Although the patient complained of partial discomfort at about 9 months after the appliance application, she showed significant improvement and stable nasality in all sentence patterns of the speech test (Nasometer II), compared to that before the appliance application. No specific findings such as nasal emission were observed; thus, an appliance reduction and removal program was carried out. At the first visit, the patient showed many errors, especially in consonants requiring oral pressure (alveolar consonants (/s/) and palatal consonants (/j/)). However, the errors in consonants almost disappeared during appliance therapy. Currently, the reduction program has been conducted four times, and the appliance has been completely removed because of consistently stable results.

## Conclusion

This case involved a patient who underwent surgery due to an incomplete cleft palate when she was a child. Even in adulthood, this patient still had articulation disorder caused by VPI. Along with speech therapy, a speech-aid prosthesis was used for treatment. Nine months after appliance application, the appliance was reduced three times. The appliance was removed after approximately 1 year and 4 months. The patient was followed up for approximately 6 months after the speech-aid appliance treatment ended. During follow-up, the test results for nasality remained normal. The patient was able to speak normally without any further surgical treatment, such as flap surgery. Although VPI treatment using a speech aid appliance is more effective when applied at a young age, if a multi-angle speech test for articulation disorder is performed, and treatment plans using speech therapy and a speech-aid prosthesis are established based on the examination, as in the current case, speech therapy using speech aids can show good results for adult patients with a history of a cleft palate. Therefore, it is hereby reported as a therapy option worthy of consideration.

## Data Availability

Not applicable

## Abbreviations

PPVT: Peabody Picture Vocabulary Test
SMCP: Submucous cleft palate
VPI: Velopharyngeal incompetence

## References

[1] Shin YJ, Ko SO (2015). Successful and rapid response of speech bulb reduction program combined with speech therapy in velopharyngeal dysfunction: a case report. Maxillofac Plast Reconstr Surg.

[2] Kuehn DP (1979). Velopharyngeal anatomy and physiology. Ear Nose Throat J.

[3] Fisher DM, Sommerlad BC (2011). Cleft lip, cleft palate, and velopharyngeal insufficiency. Plast Reconstr Surg.

[4] Kummer A (2011) Cleft palate and craniofacial anomalies: effects on speech and resonance USA. San Diego: Thomson Delmar Learning. Inc. p. 3–32.

[5] Koh K-H, Shin H-K 1992, Clinical study of velopharyngeal closure after the primary palatorrhaphy in cleft palate patients. Maxillofac Plast Reconstr Surg 14:1–21

[6] Glade RS, Deal R (2016). Diagnosis and management of velopharyngeal dysfunction. Oral Maxillofac Surg Clin.

[7] Park YH, Jo HJ, Hong IS, Leem DH, Baek JA, Ko SO (2019). Treatment of velopharyngeal insufficiency in a patient with a submucous cleft palate using a speech aid: the more treatment options, the better the treatment results. Maxillofac Plast Reconstr Surg.

[8] Ko S-O, Shin H-K (2000). Clinical assessment of the velopharyngeal incompetency speakers with speech aids. J Korean Assoc Oral Maxillofac Surg.

[9] Tachimura T, Hara H, Wada T (1995). Oral air pressure and nasal air flow rate on levator veli palatini muscle activity in patients wearing a speech appliance. Cleft Palate Craniofac J.

[10] Kipfmueller LJ, Lang BB (1972). Treating velopharyngeal inadequacies with a palatal lift prosthesis. J Prosthet Dent.

[11] Israel JM, Cook TA, Blakeley RW (1993). The use of a temporary oral prosthesis to treat speech in velopharyngeal incompetence. Facial Plast Surg.

[12] Wolfaardt JF, Wilson FB, Rochet A, McPhee L (1993). An appliance based approach to the management of palatopharyngeal incompetency: a clinical pilot project. J Prosthet Dent.

[13] Yoon B-K, Ko S-O, Shin H-K (2001). A clinical study of palatal lift for treatment of velopharyngeal incompetency. J Korean Assoc Oral Maxillofac Surg.

[14] Raju H, Padmanabhan TV, Narayan A (2009). Effect of a palatal lift prosthesis in individuals with velopharyngeal incompetence. Int J Prosthodont.

